# A Review of Current and Emerging Trends in Donor Graft-Quality Assessment Techniques

**DOI:** 10.3390/jcm11030487

**Published:** 2022-01-18

**Authors:** Natalia Warmuzińska, Kamil Łuczykowski, Barbara Bojko

**Affiliations:** Department of Pharmacodynamics and Molecular Pharmacology, Faculty of Pharmacy, Collegium Medicum in Bydgoszcz, Nicolaus Copernicus University in Torun, 85-089 Bydgoszcz, Poland; n.warmuzinska@cm.umk.pl (N.W.); k.luczykowski@cm.umk.pl (K.Ł.)

**Keywords:** kidney transplantation, graft quality assessment, biomarkers, machine perfusion, IRI, DGF

## Abstract

The number of patients placed on kidney transplant waiting lists is rapidly increasing, resulting in a growing gap between organ demand and the availability of kidneys for transplantation. This organ shortage has forced medical professionals to utilize marginal kidneys from expanded criteria donors (ECD) to broaden the donor pool and shorten wait times for patients with end-stage renal disease. However, recipients of ECD kidney grafts tend to have worse outcomes compared to those receiving organs from standard criteria donors (SCD), specifically increased risks of delayed graft function (DGF) and primary nonfunction incidence. Thus, representative methods for graft-quality assessment are strongly needed, especially for ECDs. Currently, graft-quality evaluation is limited to interpreting the donor’s recent laboratory tests, clinical risk scores, the visual evaluation of the organ, and, in some cases, a biopsy and perfusion parameters. The last few years have seen the emergence of many new technologies designed to examine organ function, including new imaging techniques, transcriptomics, genomics, proteomics, metabolomics, lipidomics, and new solutions in organ perfusion, which has enabled a deeper understanding of the complex mechanisms associated with ischemia-reperfusion injury (IRI), inflammatory process, and graft rejection. This review summarizes and assesses the strengths and weaknesses of current conventional diagnostic methods and a wide range of new potential strategies (from the last five years) with respect to donor graft-quality assessment, the identification of IRI, perfusion control, and the prediction of DGF.

## 1. Introduction

Kidney transplantation (KTx) is a life-saving treatment for patients with end-stage renal dysfunction that is characterized by higher survival rates and greater quality of patient life compared to dialysis treatment [1]. Unfortunately, the number of patients placed on kidney transplant waiting lists is rapidly increasing, resulting in a growing gap between organ demand and the availability of kidneys for transplantation. Standard criteria donors (SCD) are preferred for kidney transplants because organs from these individuals typically result in more favourable outcomes compared to other donor types [2]. However, the shortage of available kidneys has forced medical professionals to utilize marginal kidneys from expanded criteria donors (ECD) to broaden the donor pool and shorten wait times for patients with end-stage renal disease. Nonetheless, it is well known that donor organ quality affects long-term outcomes for renal transplant recipients, and ECD kidney grafts have been shown to have worse outcomes compared to SCD grafts, including an increased risk of delayed graft function (DGF) and primary nonfunction incidence (PNF) [2,3]. Thus, representative methods of assessing graft-quality are urgently needed, especially for ECDs. Currently, the surgeon decides whether to accept or decline a kidney based on their interpretation of the donor’s recent laboratory tests and a visual evaluation of the organ, with a biopsy being employed in some cases for direct tissue analysis [4,5]. Notably, the rapid emergence of techniques such as imaging, omics, and organ perfusion has provided surgeons with a wide range of new potential tools and biomarkers that could be used to evaluate graft quality.

**Figure 1:**
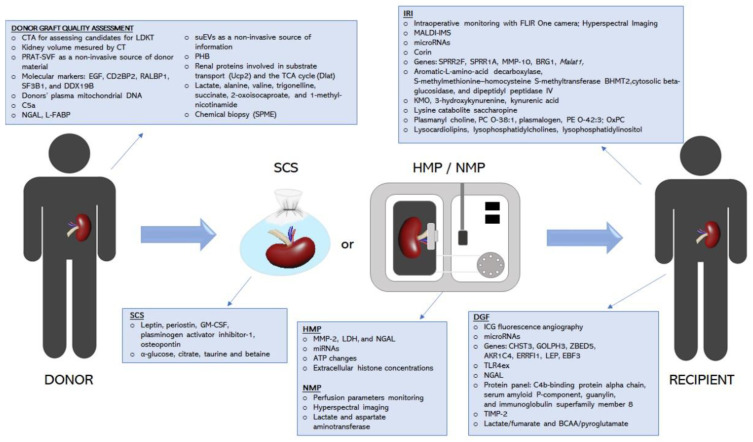
Emerging techniques and biomarkers in graft quality assessment, the identification of ischemia-reperfusion injury, perfusion control, and the prediction of DGF.

In this paper, we review and evaluate the limits and advantages of current conventional diagnostic methods and a range of new potential tools (from the last five years) with respect to donor graft-quality assessment, the identification of ischemia-reperfusion injury (IRI), perfusion control, and the prediction of DGF (Figure 1).

## 2. Current Conventional Diagnostic Methods

### 2.1. Visual Assessment

A visual evaluation of the kidney by the transplant team is a critical step in determining whether it will be accepted for transplantation or rejected. Macroscopic examination is useful for identifying kidney tumors, anatomical changes, damage, fibrosis, and scars that indicate the quality of the graft. However, this method is subjective and depends on the transplant team’s level of experience [4]. Recent findings showed that surgeons were able to reliably predict the occurrence of postperfusion syndrome through visual assessments of liver graft quality, thus emphasizing the importance of visual appraisals by the surgical team [6]. However, no prior studies have evaluated intra-observer variability and the predictive value of visual kidney assessment. Thus, there is a need for new standardized diagnostic solutions for graft-quality assessment.

### 2.2. Clinical Risk Scores

Clinical information and laboratory results for a potential donor are crucial for an initial assessment of organ quality. Consequently, several scoring systems have been created to comprehensively analyse the risk of long-term graft failure or DGF [7,8,9,10]. At present, the Kidney Donor Risk Index (KDRI) and the Kidney Donor Profile Index (KDPI) are recognized as the most effective systems for scoring kidney graft quality. The KDRI was created by Rao et al., to quantify the risk of graft failure from deceased donors (DDs) based on donor and transplant variables, such as age, serum creatinine (CR), diabetes, HCV status, and cause of death [10]. The KDPI is a percentile measure based on the KDRI that was designed to assess how long a kidney from a DD is expected to function relative to all kidneys recovered in the U.S. during the previous year. The KDPI score is calculated based on ten variably weighted donor parameters that relevantly affect organ quality, with an emphasis on nephron mass. Lower KDPI scores are linked with longer estimated organ function, while higher KDPI scores are associated with a shorter estimated organ lifespan [11,12]. The KDRI and KDPI are regarded as reliable predictors of graft outcomes, and they are expected to increase the prevalence of marginal kidney grafting and reduce the unnecessary discard rate [11,13]. However, these indexes are not intended to be used as the only metric for determining donor suitability; rather, they should be utilized as a part of a comprehensive assessment along with other factors, including pre-implant biopsy histopathology and hypothermic mechine perfusion (HMP) parameters [11,14]. Because age is the most influential factor in calculating the KDRI and KDPI scores, it is unclear whether the scores for these indexes can be applied to elderly and pediatric DDs. Recent studies suggest that the KDPI does not precisely predict pediatric kidney graft survival, while the KDRI has been found to be more reliable for elderly DDs. Overall, more research is needed to assess how reliably KDPI and KDRI scores predict postoperative renal function for grafts using kidneys from pediatric and elderly donors [13,15].

### 2.3. Biopsy

Pretransplant biopsy is currently one of the most widely used diagnostic methods and is recognized as the gold standard for confirming allograft injury. However, the frequency with which biopsies are performed varies between medical facilities and countries. In the United States, up to 85% of higher-risk kidneys are biopsied, whereas pretransplant biopsies are rarely conducted in European medical facilities. Histological evaluation is usually applied selectively, predominantly in ECD and donor after cardiac death (DCD) kidneys, and can help surgeons decide whether a kidney should be selected for transplantation or rejected [4,5,16].

In contrast to most laboratory data, histopathological assessments of biopsies do not yield a single value; rather, they produce comprehensive diagnoses that consider all available information. Although glomerulosclerosis, vascular disease, and interstitial fibrosis are the most frequently reported kidney parameters associated with worse graft outcomes [4,16], there is no consensus on the relative importance of each factor and which threshold values should be used to define the acceptable limit values. A further difficulty is the low reproducibility of kidney biopsy evaluations between on-call pathologists and renal pathologists described in many prior studies. The clear need to improve reproducibility and to objectivize the procedure and reporting of results prompted the development of several new composite histopathological scoring systems, including the Remuzzi score, the Maryland Aggregate Pathology Index, Banff criteria, and the Chronic Allograft Damage Index. Nevertheless, even with all these scoring systems, there are still doubts relating to the sampling, processing, and evaluation of biopsies [4,5,16].

In daily practice, it may be necessary to obtain quick results. In such circumstances, frozen section (FS) evaluation is often used for decision making. Producing paraffin sections (PS) is time consuming, which can cause histological evaluations to require up to 3 h to complete, even with the use of high-speed processing methods [5,17,18]. However, reports of reproducibility and prognostic value are based on paraffin-embedded tissue [18]. Recent studies have shown discrepancies in the results obtained with the use of FS and PS, but these variances had no significant impact on the outcomes for the transplanted organs [18]. Observed changes could be subtler in frozen sections than in paraffin sections, which may be a limitation, particularly in the hands of inexperienced pathologists [17,19]. On the other hand, it is also critical to consider logistics when choosing an optimal biopsy technique. For instance, FS is able to provide a diagnosis in less than 30 min, whereas PS requires at least 3 h. In selecting the proper technique, it is important to strike a balance between the benefits and risks associated with increased cold ischemia [4,18].

A lack of uniformity with respect to procedural standards has resulted in the use of a variety of biopsy techniques. The majority of medical facilities seem to prefer wedge biopsy (WB) over needle biopsy (NB) because NB carries a greater risk of injuring larger blood vessels, potentially resulting in uncontrolled bleeding after reperfusion. However, most recent reports comparing WB and NB have found that NB provides a much better evaluation of vascular lesions and has a higher overall correlation with the state of the whole kidney [5,16,17].

Ultimately, the most crucial factor is how the histopathological results correlate with long-term graft survival. Many studies have attempted to address the predictive value of renal biopsy with respect to graft outcomes, but the results of these studies have been predominantly inconclusive [20,21,22,23]. For instance, Traynor et al., conducted a retrospective study that examined kidney transplants over a 10-year period to determine whether pretransplant histology is able to predict graft outcomes at 5 years, and whether donor histology adds incremental data to the current clinical parameters. While the results of these reports suggest that that histological assessment adds little additional prognostic information aside from clinical parameters [20], Yap et al., found that the histological evaluation of ECD kidneys was associated with improved long-term graft survival. Their results suggest that pretransplant biopsy assessment can enable ECD kidneys to be used as a safe and viable option during persistent shortages of kidney donors [21]. The divergence between recent studies highlights the need for a prospective controlled trial to evaluate the predictive value of pretransplant biopsies. Until a standardized and comprehensive evaluation protocol has been developed, biopsy findings remain only one component of a donor organ assessment and should not be taken as the sole determinant in deciding whether to discard or transplant donor kidneys [19,24,25].

### 2.4. Perfusion Control

Static cold storage (SCS) and HMP are the main techniques of kidney graft preservation [26]. HMP has become a frequently and widely used procedure in kidney transplantation over the past few years [26,27,28]. Indeed, several reports have shown that the HMP reconditioning effect results in better postoperative outcomes with respect to reducing DGF and better long-term graft survival after transplantation [29,30,31]. An important benefit of HMP is that it enables the monitoring of perfusion parameters that could predict post-transplant organ viability. In particular, flow rate and renal resistance (RR) have been among the most frequently used perfusion parameters in predicting post-transplant function [27,32,33,34]. Previous studies have produced findings suggesting that real-time RR detection provides good predictive value. As Bissolati et al., showed, the RR trend during HMP can be used to predict post-transplantation outcomes, especially in relation to kidneys procured from ECD [28]. Patel et al., conducted a retrospective study that included 190 kidneys in order to evaluate the prognostic utility of HMP in DD transplantation. Their findings showed that resistances at two hours and beyond predicted DGF, while initial resistance to machine perfusion predicted one-year graft survival post-transplantation [35]. On the other hand, some studies found no association between hemodynamic parameters during HMP and the development of DGF [27]. Thus, due to these inconclusive results, the perfusion parameters cannot be regarded as stand-alone criteria. However, the undoubted advantage of perfusion parameters is that they are easy to obtain in a non-invasive manner. As such, Jochmans et al., and Zheng et al., have suggested that HMP parameters should be included as part of a comprehensive graft assessment [14,32]. DGF has a complex pathogenesis and cannot be predicted with precision using the HMP parameters as a stand-alone assessment tool. However, RR represents an additional source of information that can help clinicians in their decision-making process. Attaining more accurate predictions of graft outcomes will require integrating the perfusion parameters into multifactorial graft quality scoring systems. A combination of the donor’s clinical data, kidney pre-implant histopathology, and HMP parameters may provide a more effective prediction of DGF than any of the measures alone [14,32].

### 2.5. Microbiological Analysis of Preservation Fluid

Organ transplant recipients are prone to infectious complications, and despite many advances, post-operative infections remain associated with significant morbidity and mortality [36,37,38]. Early post-transplant infections among kidney transplant recipients may be transmitted via the donor, or the donated organ may be contaminated during the transplantation procedure [36,38]. Moreover, pathogens can be transmitted via preservation solution, which is required to maintain kidney viability, but due to its biochemical characteristics, it can also keep microorganisms alive and serve as an infection vector [36,38,39]. For that reason, some transplant centres collect preservation fluid for microbiological analysis in addition to standard screening for donor infections. However, there are no widely accepted recommendations for managing positive preservation fluid cultures [36,38]. Moreover, it remains unanswered whether intra-operative preservation fluid routine screening should be performed because the clinical impact of this practice is still not well established. Some studies have evaluated the risk factors associated with culture-positive preservation fluid and determined the benefit of routine screening of preservation solutions for the management of kidney transplant recipients [36,37,38,40]. Corbel et al., demonstrated that 24% of DD preservation fluid cultures were positive, and these contaminations were mainly a consequence of procurement procedures [37]. Reticker et al. [36] and Oriol et al. [38] showed that the prevalence of culture-positive preservation fluid was up to 60%; however, the vast majority of microbial growth was consistent with skin flora or low-virulence pathogens. In addition, Oriol et al., indicated that pre-emptive antibiotic therapy for recipients with high-risk culture-positive preservation fluid might improve the outcomes and help to avoid preservation-fluid-related infections [38]. Moreover, Stern et al., reported that fungal contamination of preservation fluid was infrequent, although yeast contamination of preservation solutions was associated with high mortality [40]. In parallel, Reticker et al., suggested that antibiotic therapy for recipients with preservation solutions contaminated by low virulence pathogens may not be necessary, reducing antibiotic overuse [36]. In conclusion, routine screening of preservation solutions could improve graft outcomes and pre-emptive antibiotic therapy and be helpful to avoid preservation-fluid-related infections. However, future studies are needed to establish guidelines for preservation fluid microbiological analysis and handling culture-positive preservation fluid.

## 3. Emerging Techniques

### 3.1. Imaging

Diagnostic imaging methods are mainly used to evaluate kidneys from living donors (LD) prior to acceptance for transplantation, as well as for assessing post-renal transplant complications. In the case of living donor surgeries, non-invasive preoperative evaluation of the quality of the graft organ is especially critical, which allows surgeons to assess certain vital features, such as size, the presence/absence of focal cystic or solid lesions, and the condition of vascular structures, to establish whether it is appropriate for transplantation. While most of these features can be visualized via Doppler ultrasound, computed tomography angiography (CTA) is usually necessary for a more accurate assessment of the vascular anatomy [41,42,43]. However, given the critical role of careful evaluation and suitable preparation when dealing with living donor transplantation, it will be imperative to continue to conduct new research aimed at improving transplantation outcomes. 

Sarier et al., conducted a retrospective study wherein they compared pretransplant CTA images to intraoperative findings to evaluate renal artery variations in a large sample of LD. They found that laparoscopic donor nephrectomy enabled the detection of the same number of renal arteries as CTA in 97.9% of the analysed kidneys, but less than CTA in the remaining 2.1%. Notably, a greater number of renal arteries were not detected in any of the studied kidneys via nephrectomy compared to CTA. These results indicate that CTA is more accurate than intraoperative findings, and is an effective method for evaluating candidate donors for living donor kidney transplantation (LDKT), as well as for identifying renovascular variations [42].

Al-Adra et al., employed computed tomography (CT) scans to assess the influence of donor kidney volume on recipient estimated glomerular filtration rate (eGFR) in a large cohort of patients undergoing LDKT. The resultant statistical models showed a significant correlation between donor kidney volume and recipient eGFR at 1, 3, and 6 months (*p* < 0.001). These findings indicate that donor kidney volume is a strong independent predictor of recipient eGFR in LDKT and may therefore be a valuable addition to predictive models of eGFR after transplantation. Further research could examine whether addition of donor kidney volume in matching algorithms can improve recipient outcomes [43].

Although the ability to monitor graft status intraoperatively is limited at present, several novel solutions have been proposed over the past few years to evaluate graft quality during transplantation and predict DGF.

In 2019, Fernandez et al., proposed a novel approach that utilized infrared imaging to monitor the reperfusion phase during kidney transplantation in real-time. To this end, they used a long-wave infrared camera (FLIR One) with a visual resolution of 1440 × 1080 pixels and a thermal resolution of 160 × 120 to study the grafts in 10 pediatric patients undergoing kidney transplantation. During the study, images were acquired at several key time points. The authors observed a correlation between changes in intraoperative graft temperature and decreases in postoperative creatinine levels in all of the analysed subjects. Given these results, Fernandez et al., concluded that infrared thermal imaging could be a promising option for non-invasive graft perfusion monitoring. However, additional work is required to confirm Fernandez et al.’s results because they were somewhat limited due to the relatively small number of patients included and the short follow-up period [44].

In another study, Sucher et al., employed Hyperspectral Imaging (HSI) as a noncontact, non-invasive, and non-ionizing method of acquiring quantitative information relating to kidney viability and performance during transplantation. Specifically, they used HSI to study seventeen consecutive deceased donor kidney transplants prior to transplantation, while stored on ice, and again at 15 and 45 min after reperfusion. After computation time of less than 8 s, the analysis software was able to provide an RGB image and 4 false color images representing the physiological parameters of the recorded tissue area, namely, tissue oxygenation, perfusion, organ hemoglobin, and tissue water index. The obtained results revealed that allograft oxygenation and microperfusion were significantly lower in patients with DGF. Future applications might also utilize HSI during donor surgery to assess kidney quality prior to cold perfusion and procurement. However, HSI can only be used intraoperatively and requires a direct view of the kidney because the maximum penetration depth for microcirculation measurements is currently 4–6 millimetres, making transcutaneous applications impossible. Thus, this technique’s main limitations are its inability to provide continuous or intermittent transcutaneous follow-up measurements, as well as its small sample size. Thus, further studies are required to confirmed these results [45].

In the recent article, Gerken et al., documented a prospective diagnostic study that they had conducted in two German transplantation centres wherein allograft microperfusion was assessed intraoperatively via near-infrared fluorescence angiography with indocyanine green (ICG). While previous studies have shown that ICG fluorescence angiography can be applied safely during kidney transplantation, none have provided a quantitative assessment of the use of fluorescence video. To fill this gap, Gerken et al., evaluated the benefits of coupling quantitative intraoperative fluorescence angiography with ICG to predict post-operative graft function and the occurrence of DGF. Their findings indicated that the impairment of intraoperative microperfusion in the allograft cortex is a risk factor for the occurrence of DGF, and that ICG Ingress is an independent predictor of DGF. Further studies are warranted to analyse the effect of applying early therapeutic approaches to prevent DGF in kidney transplant recipients, thus improving long-term graft success [46].

The use of imaging techniques to diagnose post-renal transplant complications has been discussed extensively in recent reviews [47,48,49]; therefore, the present work will only examine a few of the most recent studies in this field. Promising results have been reported with respect to combining positron emission tomography (PET) with CT or magnetic resonance imaging (MRI) using the glucose analogue radiotracer, 2-deoxy-2-fluoro-D-glucose (FDG), to detect acute kidney allograft rejection, for diagnostic applications, for the functional assessment of grafts, and for therapeutic monitoring [50,51]. In another study, the utility of arterial spin labeling (ASL) magnetic resonance imaging was evaluated for its ability to identify kidney allografts with underlying pathologies. ASL uses endogenous water as a tracer, and it has previously been used in applications relating to the brain. Moreover, there have been reports demonstrating that ASL can be used to categorize stages of chronic kidney disease [52]. Wang et al., demonstrated that ASL might be a non-invasive tool for differentiating kidneys with subclinical pathology from those with stable graft function. However, more research should be performed to verify these findings [53].

### 3.2. Omics

The last few years has seen the emergence of many new technologies that examine organ function on a molecular level, which has enabled the discovery of numerous potential biomarkers of renal injury. High-throughput omics technologies allow researchers to obtain a large amount of data about specific types of molecules, providing a holistic picture that captures the complex and dynamic interactions within a biological system. These innovative methods, including transcriptomics, genomics, proteomics, metabolomics, and lipidomics, provide a deeper understanding of the complex mechanisms associated with IRI, inflammatory processes, and graft rejection [5,54]. This section surveys some promising methods and techniques that could be successfully translated to clinical settings in the foreseeable future (Table 1).

#### 3.2.1. Transcriptomics/Genomics

Several studies have examined how graft quality and donor category impact graft and patient survival. Giraud et al., proposed an open-ended approach based on microarray technology to understand IRI occurring in DCD kidneys in a preclinical porcine model that had been subjected to warm ischemia (WI) followed by cold ischemia. Giraud et al.’s findings indicated that hundreds of cortex and corticomedullary junction genes were significantly regulated after WI or after WI followed by cold storage compared to healthy kidneys. In addition, they also analysed the kinetics of the most differentially expressed genes. They hypothesized that these genes played a key role in IRI and could be divided into eight categories: mitochondria and redox state regulation; inflammation and apoptosis; and protein folding and proteasome; cell cycle, cellular differentiation and proliferation; nucleus genes and transcriptional regulation; transporters; metabolism regulation; mitogen-activated protein kinase and GTPase (guanosine triphosphate, GTP) activity [55].

Boissier et al., performed a comparative study of cellular components, transcriptomics, and the vasculogenic profiles obtained from 22 optimal donors and 31 deceased ECDs. They hypothesized that as an easily accessible source of donor-derived material, perirenal adipose tissue (PRAT) can be used to assess the quantitative and functional features that characterize donor cells. In addition, adipose tissue can be enzymatically processed to obtain stromal vascular fraction (SVF), which is a heterogeneous cellular mixture free of adipocytes. In their study, Bossier et al., performed a transcriptomic analysis in order to differentiate the PRAT-SVF molecular transcript in ECD and other donors. The upregulated genes demonstrated a strong association with the inflammatory response, cytokine secretion, and circulatory system development, while the downregulated genes were associated with regulating metabolic processes and circulatory system development. Importantly, Bossier et al.’s findings provide new evidence that PRAT-SVF serves as a non-invasive source of donor material that can be highly valuable in the assessment of inflammatory features affecting the quality and function of the graft [56].

The midterm outcomes of kidney transplant recipients with early borderline changes between ECD, SCD, and LD were compared in a retrospective observational study. In the ECD group, microarray analysis showed a higher expression of 244 transcripts than the SCD group, and 437 more than the LD group. Compared to both the SCD and LD groups, gene annotation analysis of transcripts with elevated expression in ECD group revealed enhancement in the inflammatory response, the response to wounding, the defence response, and the ECM-receptor interaction pathway. ECD-related transcripts were likely increased by already occurred vascular changes compared to SCD group, and, similarly in SCD group, by longer ischemia compared with LD group. Therefore, chronic vascular changes and cold ischemia time enhance inflammation and thus contribute to poor outcomes for these grafts [57].

Another novel organ-evaluation tool was proposed in a retrospective open-cohort study that examined donors’ plasma mitochondrial DNA (mtDNA), which can be easily and non-invasively assayed in the pre-transplant period, and may be a promising predictive biomarker for allograft function [58]. The mtDNA levels in the plasma of DCD were determined via real-time polymerase chain reaction (RT-PCR) and then statistically analysed in relation to the recipient’s mtDNA levels and DGF. The linear prediction model, which included plasma mtDNA, donor serum creatinine, and warm ischemia time (WIT), showed high predictive value for reduced graft function. Moreover, the findings indicated that plasma mtDNA might be a novel non-invasive predictor of DGF and allograft function at six months after transplantation, in addition to correlating to allograft survival. Furthermore, mtDNA may serve as a surrogate predictive marker for PNF [58].

The vast majority of studies aiming to identify novel biomarkers involved in IRI have used murine or rat models. A growing body of evidence indicates that the aberrant expression of microRNAs (miRNA/miR) is closely associated with IRI pathogenesis [59,60,61,62,63,64]. MiRNAs are small, noncoding RNAs that mediate mRNA cleavage, translational repression, or mRNA destabilization [59]. For instance, Chen et al.’s findings suggest that miR-16 may serve as a potential biomarker of IRI-induced acute kidney injury (AKI) [59], while Zhu et al., found that miR-142-5p and miR-181a might be responsible for modulating renal IRI development [63]. On the other hand, some studies have pointed that miR-17-92, miR-139-5p, and miR-27a may play a protective role in IRI [61,62,64]. For example, Song et al., suggest that the overexpression of miR-17-92 could partly reverse the side-effects of IRI on the proximal tubules in vivo [61]. Furthermore, Wang et al., have reported that the overexpression of miR-27a results in the downregulation of toll-like receptor 4 (TLR4), which in turn inhibits inflammation, cell adhesion, and cell death in IRI [62].

Other murine-model-based studies have explored new candidate genes associated with renal IRI. In one such study, Su et al., found that IRI caused the upregulation of SPRR2F, SPRR1A, MMP-10, and long noncoding RNA (lncRNA) *Malat1* in kidney tissues. These genes are involved in keratinocyte differentiation, regeneration, and the repair of kidney tissues; extracellular matrix degradation and remodeling; inflammation; and cell proliferation in renal IRI [64]. In a separate study, Liu et al., investigated the role of BRG1 in IRI-induced AKI with a focus on its role in regulating IL-33 expression in endothelial cells. Their findings revealed that endothelial BRG1 deficiency reduces renal inflammation following ischemia-reperfusion in mice with a simultaneous reduction in IL-33 levels [65].

Comparisons of IRI in murine-based models and clinical studies have yielded valuable results [66,67]. For instance, Cippà et al., employed RNA-sequencing-mediated transcriptional profiling and machine learning computational approaches to analyse the molecular responses associated with IRI, which emphasized early markers of kidney disease progression and outlined transcriptional programs involved in the transition to chronic injury [66]. Other studies have demonstrated that Corin is downregulated in renal IRI and may be associated with DGF after kidney transplantation. Researchers have also screened differentially expressed genes in a murine model of IRI, with findings identifying Corin as one of the most relevant downregulated genes among 2218 differentially expressed genes. Moreover, 11 recipients with complications due to DGF and 16 without DGF were recruited for an ELISA to determine their plasma Corin concentrations. The findings of this study showed downregulation of plasma Corin concentrations in transplant recipients with DGF complications, indicating that Corin could be a potential biomarker of DGF [67]. DGF may result from early ischemic injury and potentially contribute to poor long-term survival following kidney transplantation [68,69]. For this reason, much research has been devoted to devising reliable methods for predicting the extent of IRI, and hence, DGF.

Hence, as with the IRI, miRNA was evaluated as a biomarker of DGF. In one study, Khalid et al., quantified microRNAs in urine samples from kidney transplant patients to determine whether this approach can be used to predict who will develop DGF following kidney transplantation. To this end, they used unbiased profiling to identify microRNAs that are predictive of DGF following kidney transplantation (i.e., miR-9, -10a, -21, -29a, -221, and -429), and afterward confirmed their findings by measuring specific microRNAs via RT-PCR. The biomarker panel was then assessed using an independent cohort at a separate transplant centre, with urine samples being collected at varying times during the first week after transplantation. When considered individually, all miRs in the panel showed a trend towards an increase or relevant increase in patients with DGF [68].

Wang et al., used high-throughput sequencing to investigate the miRNA expression profiling of exosomes in the peripheral blood of kidney recipients with and without DGF, and explain the regulation of miRNAs in the DGF pathogenesis [69]. Exosomes are cell-derived membrane vesicles present in numerous bodily fluids that play a crucial role in processes such as the regulation of cellular activity, intercellular communication, and waste management [69,70]. Wang et al., identified 52 known and 5 conserved exosomal miRNAs specifically expressed in transplant recipients with DGF. Additionally, their findings showed that transplant recipients with DGF also exhibited the upregulation of three co-expressed miRNAs: hsa-miR-33a-5p R-1, hsa-miR-98-5p, and hsa-miR-151a-5p. Moreover, hsa-miR-151a-5p was positively correlated with the kidney recipients’ serum CR, blood urea nitrogen (BUN), and uric acid (UA) levels in the first week post-transplantation [69].

MicroRNA expression in kidney transplant recipients with DGF has also been assessed in another recently published study [71]. In this work, the researchers employed RT-PCR to analyse the expression of miRNA-146-5p in peripheral blood and renal tissue obtained from kidney transplant recipients who had undergone a surveillance graft biopsy during the DGF period. In the renal tissue, the expression of miR-146a-5p was significantly increased among the DGF patients compared to the stable and acute rejection (AR) patients. Similarly, microRNA 146a-5p had heightened expression in the peripheral blood samples from the DGF group compared to those of the acute rejection and stable groups; however, these differences were not statistically significant (*p* = 0.083) [71].

Overall, all these reports indicate that miRNAs are emerging as essential biomarkers in the molecular diagnosis of DGF. The above-discussed findings identify biomarkers that could contribute to the development of tools for predicting DGF and, as such, represent an important area of focus for future research.

Zmonarski et al., applied PCR to nonstimulated peripheral blood mononuclear cells (PBMCs) to examine the averaged mRNA toll-like receptor 4 expression (TLR4ex). The sample for this study consisted of 143 kidney transplant patients, 46 of whom had a history of DGF, and a control group of 38 healthy volunteers. The patients with a history of DGF were divided into two subgroups based on the median TLRex: low-TLR4 expression and high-TLR expression. Zmonarski et al.’s findings showed that patients with DGF had a much lower TLR4ex and worse parameters of kidney function. In addition, while a comparison of the DGF patients with low and high TLR4ex revealed no initial differences in kidney transplant function, differences were observed in the post-follow-up period. Furthermore, regression analysis showed that TLR4ex was related to recipient age, tacrolimus concentration, and uremic milieu. Consequently, the authors concluded that the low TLR4 expression in patients with DGF may be associated with poor graft-capacity prognosis, and that analysis of changes in TLR4ex may be valuable for assessing immunosuppression efficacy [72]. 

Another study aiming to identify potential biomarkers of DGF and AKI was recently conducted by Bi et al. [73]. In this study, the authors obtained two mRNA expression profiles from the National Center of Biotechnology Information Gene Expression Omnibus repository, including 20 DGF and 68 immediate graft function (IGF) samples. Differentially expressed genes (DEGs) in the DGF and IGF groups were identified, and pathway analysis of these DEGs was conducted using the Gene Ontology and Kyoto Encyclopedia of Genes and Genomes. Next, a protein–protein interaction analysis extracted hub genes. The essential genes were then searched in the literature and cross-validated based on the training dataset. In total, 330 DEGs were identified in the DGF and IGF samples, including 179 upregulated and 151 downregulated genes. Of these, OLIG3, EBF3, and ETV1 were transcription factor genes, while LEP, EIF4A3, WDR3, MC4R, PPP2CB, DDX21, and GPT served as hub genes in the PPI network. In addition, the findings suggested that EBF3 may be associated with the development of AKI following renal transplantation because it was significantly upregulated in the validation dataset (GSE139061), which is consistent with the initial gene differential expression analysis. Moreover, the authors found that LEP had a good diagnostic value for AKI (AUC = 0.740). Overall, these findings provided more profound insights into the diagnosis of AKI following kidney transplantation [73].

Elsewhere, McGuinness et al., combined epigenetic and transcriptomic data sets to determine a molecular signature for loss of resilience and impaired graft function. Notably, at a translational level, this study also provided a platform for developing a universal IRI signature and the ability to link it to post-transplant outcomes. Furthermore, McGuinness et al.’s findings relate DNA methylation status to reperfusion injury and DGF outcome. In this study, 24 paired pre- and post-perfusion renal biopsies defined as either meeting the extreme DGF phenotype or exhibiting IGF were selected for analysis. The findings of this analysis showed that the molecular signature contained 42 specific transcripts, related through IFNγ signaling, which, in allografts displaying clinically impaired function (DGF), exhibited a major change in transcriptional amplitude and increased expression of noncoding RNAs and pseudogenes, which is consistent with increased allostatic load. This phenomenon was attended by an increase in DNA methylation within the promoter and intragenic regions of the DGF panel in pre-perfusion allografts with IGF. Overall, McGuinness et al.’s findings suggest that kidneys exhibiting DGF suffer from an impaired ability to restore physiological homeostasis in response to stress that is commensurate to their biological age and associated allostatic load. This outcome is reflected in changes in the epigenome and transcriptome, as well as in the dysregulation of RNA metabolism [3].

#### 3.2.2. Proteomics

Proteomics approaches have also been used to identify donor biomarkers that may predict graft dysfunction in order to alleviate organ shortages and address the lack of representative methods for assessing graft quality. To date, several studies have focused on identifying novel proteomic biomarkers of graft quality in donor urine [74,75,76,77]. Koo et al.’s study aimed to investigate the viability of using the levels of neutrophil gelatinase-associated lipocalin (NGAL), kidney injury molecule-1 (KIM-1), and L-type fatty acid binding protein (L-FABP) in donor urine samples to predict reduced graft function (RGF). In addition, Koo et al., also created a prediction model of early graft dysfunction based on these donor biomarkers. This model, which includes donor urinary NGAL, L-FABP, and serum CR, has been shown to provide better predictive value for RGF than donor serum CR alone. Based on this model, a nomogram for a scoring method to predict RGF was created to help guide the allocation of DD and maximize organ utilization [74]. On the other hand, another large prospective study has shown that donor injury biomarkers such as microalbumin, NGAL, KIM-1, IL-18, and L-FABP have limited utility in predicting outcomes among kidney transplant recipients [75]. This study evaluated the associations between injury biomarkers in the urine of DD and donor AKI, recipient DGF, and recipient six-month eGFR. Each of the tested biomarkers was strongly associated with donor AKI in the adjusted analyses. However, although the levels of all five donor biomarkers were higher in recipients with DGF than in those without DGF, the fully adjusted analyses revealed an association between higher donor urinary NGAL concentrations and a modest increase in the relative risk of recipient DGF. Moreover, the results of this study indicated that donor urinary biomarkers add minimal value in predicting recipient allograft function at six months post-transplantation [75]. In both studies, the tested biomarkers were strongly associated with donor AKI, while NGAL concentration was associated with DGF. A potential explanation for the different conclusions of these studies may be that Koo et al., used RGF as an outcome in their study, while Reese et al., used DGF due to different donor characteristics. Furthermore, it is worth emphasizing that, while these proteins are upregulated and secreted in urine in response to tubular injury, they were reported to have low specificity for tubular epithelial cell injury and were observed to increase in patients with urinary tract infections and sepsis [78,79].

In another study, the potential utility of C3a and C5a in DD urine samples as biomarkers for early post-transplant outcomes was investigated [76]. The results of this large, prospective, observational cohort study indicated a three-fold increase in C5a concentrations in urine samples from donors with stage 2 and 3 AKI compared to donors without AKI. In addition, donor C5a was positively correlated with the occurrence of DGF in recipients. In adjusted analyses, C5a remained independently correlated with recipient DGF only for donors without AKI. Moreover, the authors observed a tendency to indicate better 12-month organ functioning from donors with the lowest urinary C5a [76]. 

Monocyte chemoattractant protein-1 (MCP-1) has also been proposed as a potential biomarker of donor kidney quality. For example, Mansour et al., evaluated the association between graft outcomes and levels of MCP-1 in urine from DD at the time of organ procurement. In particular, they measured MCP-1 concentration to determine its correlation to donor AKI, recipient DGF, six-month estimated eGFR, and graft failure. Unfortunately, Mansour et al.’s results suggested that urinary MCP-1 has minimal clinical utility. Although median urinary MCP-1 concentrations were elevated in donors with AKI compared to those without AKI, higher MCP-1 levels were independently associated with a higher six-month eGFR in those without DGF. However, MCP-1 was not independently associated with DGF, and no independent associations between MCP-1 and graft failure were observed over a median follow-up of ~two years [77].

Recently, Braun et al., demonstrated the potential of using small urinary extracellular vesicles (suEVs) as a non-invasive source of data regarding early molecular processes in transplant biology. Their unbiased proteomic analysis revealed temporal patterns in the signature of suEV proteins, as well as cellular processes involved in both early response and longer-term graft adaptation. In addition, a subsequent correlative analysis identified potential prognostic markers of future graft function, such as phosphoenol pyruvate carboxykinase (PCK2). However, while Braun et al.’s study showed the potential of suEVs as biomarkers, the small number of patients in their sample did not allow for a conclusive statement on the predictive value of suEV PCK2. Therefore, the potential use of this biomarker will depend on larger trials in the future [80]. 

Studies focusing on the use of kidney tissue as a sample matrix to evaluate donor organ quality have also been performed. Using a rabbit model of brain death (BD), Li et al., employed two-dimensional gel electrophoresis and Matrix Assisted Laser Desorption/Ionization Time-of-Flight Mass Spectrometry (MALDI-TOF-MS)-based comparative proteomic analysis to profile the differentially-expressed proteins between BD and renal tissue collected from a control group. The authors were able to acquire five downregulated proteins and five upregulated proteins, which were then classified according to their function, including their association with proliferation and differentiation, signal transduction, protein modification, electron transport chain, and oxidation-reduction. Moreover, immunohistochemical analysis indicated that the expression of prohibitin (PHB) gradually elevated in a time-dependent manner. These data showed alterations in the levels of certain proteins in the organs from the BD group, even in the case of non-obvious functional and morphological changes. Given their results, Li et al., suggested that PHB may be an innovative biomarker for the primary assessment of the quality of kidneys from BD donors [81]. 

Conversely, van Erp et al., used a multi-omics approach and a rat model to investigate organ-specific responses in the kidneys and liver during BD. The application of proteomics analysis enabled them to quantify 50 proteins involved in oxidative phosphorylation, tricarboxylic acid (TCA) cycle, fatty acid oxidation (FAO), substrate transport, and several antioxidant enzymes in isolated hepatic and renal mitochondria. The most relevant changes were observed in the reduced peptide levels in the kidneys, which were related to complex I (Ndufs1), the TCA cycle (Aco2, Fh, and Suclg2), FAO (Hadhb), and the connection between FAO and the electron transport chain (Etfdh). The expression of two renal proteins, which were associated with substrate transport (Ucp2) and the TCA cycle (Dlat), was significantly increased in samples from the BD group compared to the sham-operated group. Interestingly, van Erp et al.’s findings showed that BD pathophysiology affects systemic metabolic processes, alongside organ-explicit metabolic changes, manifest in the kidneys by metabolic shutdown and suffering from oxidative stress, and a shift to anaerobic energy production, while kidney perfusion decreases. Ultimately, van Erp et al., concluded that an organ-specific strategy focusing on metabolic changes and graft perfusion should be part of novel procedures for assessing graft quality in organs from brain-dead donor, and may be the key to improving transplantation outcomes [82].

The vast majority of studies focusing on IRI have used animal models. In one proteo-metabolomics study using rat models, coagulation, complement pathways, and fatty acid (FA) signaling were observed following the elevation of proteins belonging to acute phase response due to IRI. Moreover, after 4 h of reperfusion, analysis of metabolic changes showed an increase in glycolysis, lipids, and FAs, while mitochondrial function and adenosine triphosphate (ATP) production were impaired after 24 h [83]. The authors of another study that used a porcine model of IRI found that integrative proteome analysis can provide a panel of potential—and predominantly renal—biomarkers at many levels, as changes occurring in the tissue are reflected in serum and urine protein profiles. This conclusion was based on the use of urine, serum, and renal cortex samples. In the renal cortex proteome, the authors observed an elevation in the synthesis of proteins in the ischemic kidney (vs. the contralateral kidney), which was highlighted by transcription factors and epithelial adherens junction proteins. Intersecting the set of proteins up- or downregulated in the ischemic tissue with both serum and urine proteomes, authors identified six proteins in the serum that may provide a set of targets of kidney injury. In addition, four urinary proteins with predominantly renal gene expression were also identified: aromatic-L-amino-acid decarboxylase (AADC), S-methylmethionine–homocysteine S-methyltransferase BHMT2 (BHMT2), cytosolic beta-glucosidase (GBA3), and dipeptidyl peptidase IV (DPPIV) [84]. Recent research by Moser et al., has examined kidney preservation injury and the nephroprotective activity of doxycycline (Doxy). In this work, rat kidneys were cold perfused with and without Doxy for 22 h, followed by the extraction of proteins from the renal tissue. Subsequent analysis showed a significant difference in eight enzymes involved in cellular and mitochondrial metabolism. Interestingly, the levels of N(G),N(G)-dimethylarginine dimethylaminohydrolase and phosphoglycerate kinase 1 decreased during cold perfusion on its own but increased during cold perfusion with Doxy [85]. The influence of perfusion type on graft quality has also been evaluated by Weissenbacher et al., who applied proteomics analysis to determine the differences between normothermically perfused (normothermic machine perfusion, NMP) human kidneys with urine recirculation (URC) and urine replacement (UR). Their findings revealed that damage-associated patterns in the kidney tissue decreased after 6 h of NMP with URC, suggesting decreased inflammation. Furthermore, they also observed that vasoconstriction in the kidneys was also attenuated with URC, as indicated by a reduction in angiotensinogen levels. The kidneys became metabolically active during NMP, which could be improved and prolonged by applying URC. The application of URC also enhanced mitochondrial succinate dehydrogenase enzyme levels and carbonic anhydrase, which contributed to pH stabilization. Key enzymes involved in glucose metabolism increased after 12 and 24 h of NMP with URC, including mitochondrial malate dehydrogenase and glutamic-oxaloacetic transaminase, predominantly in DCD tissue. The authors concluded that NMP with URC can prolong organ preservation and revitalize metabolism to possibly better mitigate IRI in discarded kidneys [86].

Ischemic injury may result in DGF, which is associated with a more complicated post-operative course, including a higher risk of AR [87]. Therefore, the early evaluation of kidney function following transplantation is essential for predicting graft outcomes [88]. Several studies have applied proteomic analysis to recipient urine samples in an attempt to identify protein biomarkers of DGF [87,88,89]. For instance, Lacquaniti et al., evaluated the usefulness of NGAL levels both for the early detection of DGF and as a long-term predictor of graft outcome. Their findings revealed that serum and urine samples from DGF patients contained high levels of NGAL beginning the first day after transplantation. Moreover, in patients who had received a kidney from a living related donor with excellent allograft function, NGAL concentrations lowered quickly during the first 24 h post-transplant period, reflecting a more pronounced reversible short-term injury. Importantly, NGAL levels in urine provided a better diagnostic profile than serum NGAL. Hence, urinary biomarkers on day 1 post-transplant may not only be useful in predicting who will need dialysis within one week, but they may also allow clinicians to discriminate between more subtle allograft recovery patterns [88]. However, as mentioned above, NGAL is characterized by low specificity; hence, its clinical application is limited due to inconclusive results [78,79]. Williams et al., used a Targeted Urine Proteome Assay (TUPA) to identify biomarkers of DGF following kidney transplantation. After employing data quality consideration and rigorous statistical analysis, they identified a panel of the top 4 protein biomarkers, including the C4b-binding protein alpha chain, serum amyloid P-component, guanylin, and immunoglobulin superfamily member 8, which had an AUC of 0.891, a specificity of 82.6%, and a sensitivity of 77.4% [87]. Similarly, urinary tissue inhibitor of metalloproteinases-2 (TIMP-2) and insulin-like growth factor binding protein-7 (IGFBP7) have been evaluated as biomarkers for DGF [89]. The findings of these studies indicated that TIMP-2 was able to adequately identify patients with DGF and prolonged DGF (AUC 0.89 and 0.77, respectively), whereas IGFBP7 was not. Moreover, correcting TIMP-2 for urine osmolality improved predictability (AUC 0.91 for DGF, AUC 0.80 for prolonged DGF), and 24-h urinary CR excretion and TIMP-2/mOsm were found to be significant predictors of DGF, with an AUC of 0.90. Hence, the obtained results indicated that TIMP-2 might be a promising, non-invasive indicator for predicting the occurrence and duration of DGF in individual patients [89].

#### 3.2.3. Metabolomics and Lipidomics

In the absence of good quantitative biomarkers correlating to pre-transplantation organ quality, van Erp et al., examined metabolic alterations during BD using hyperpolarized magnetic resonance (MR) spectroscopy and ex vivo graft glucose metabolism during normothermic isolated perfused kidney (IPK) machine perfusion [90]. To this end, they employed hyperpolarized ^13^C-labeled pyruvate MR spectroscopy to quantify pyruvate metabolism in the kidneys and liver at three time points during BD in a rat model. Following BD, glucose oxidation was measured using tritium-labeled glucose (_D_-6-3H-glucose) during IPK reperfusion. In addition, enriched ^13^C-pyruvate was injected repetitively to evaluate the metabolic profile at T = 0, T = 2, and T = 4 h via the relative conversion of pyruvate into lactate, alanine, and bicarbonate. The rats showed significantly higher lactate levels immediately following the induction of BD, with alanine production decreasing in the kidneys 4 h post-BD. However, it should be emphasized that this study’s results did not assess whether these metabolic alterations can be associated with graft quality, or if they are suitable predictors of transplant outcome [90]. 

Another study using a rodent model of IRI examined the potential of using Hyperpolarized ^13^C-labeled pyruvate to evaluate the metabolic profile directly in the kidneys [91]. The in vivo responses observed at 24 h and 7 d following ischemic injury demonstrated a similar trend towards a general decrease in the overall metabolism in the ischemic kidney and a compensatory increase in anaerobic metabolism, which is evidenced by elevated lactate production, compared to aerobic metabolism. In addition, a correlation was found between the intra-renal metabolic profile 24 h after reperfusion and 7 d after injury induction, as well as a correlation with the plasma CR. As a result, the authors suggest that using hyperpolarized ^13^C-labeled pyruvate to identify the balance between anaerobic and aerobic metabolism has great future potential as a prognostic biomarker [91]. 

Increased lactate levels due to IRI were also observed in another study [92]. However, analysis of urine samples via nuclear magnetic resonance (NMR) spectroscopy showed higher levels of valine and alanine and decreased levels of metabolites such as trigonelline, succinate, 2-oxoisocaproate, and 1-methyl-nicotinamide following IRI, which was likely due to altered kidney function or metabolism [92]. 

A novel and minimally invasive metabolomic and lipidomic diagnostic protocol based on solid-phase microextraction (SPME) has been proposed to address the lack of representative methods of assessing graft quality [93,94]. The small size of the SPME probe allows the performance of chemical biopsy, which enables metabolites to be extracted directly from the kidney without any tissue collection. Furthermore, SPME’s minimally invasive nature permits multiple analyses over time. For instance, ischemia-induced alterations in the metabolic profile of the kidneys and oxidative stress as a function of cold storage were observed in one study that used an animal model, with the most pronounced alterations being observed in the levels of essential amino acids and purine nucleosides [93]. However, more work is required to discriminate a set of characteristic compounds that could serve as biomarkers of graft quality and indicators of possible development of organ dysfunction. 

In response to reports that the pharmacological inhibition of kynurenine 3-monooxygenase (KMO), and, separately, the transcriptional blockage of the Kmo gene, reduces 3-hydroxykynurenine formation and protects against secondary AKI, Zheng et al., investigated whether mice lacking functional KMO (*Kmo*^null^ mice) are protected from AKI experimentally induced by the direct induction of renal IRI [95]. KMO plays a crucial role in kynurenine metabolism. Kynurenine metabolites are generated by tryptophan catabolism and are involved in the regulation of various biological processes, including host-microbiome signaling, immune cell response, and neuronal excitability. The kynurenine pathway diverges into two distinct branches, which are regulated by kynurenine aminotransferases (KATs) and KMO, respectively. KMO is the only route of 3-hydroxykynurenine production that is known to be injurious to cells and tissue. Kynurenine may also be metabolized into kynurenic acid by KATs and to anthranilic acid by kynureninase [95]. Following the experimental induction of AKI via renal IRI, Zheng et al., observed that the *Kmo^null^* mice had kept renal function, decreased renal tubular cell injury, and fewer infiltrating neutrophils than the wild-type control mice. Given these results, they suggested that KMO is a critical regulator of renal IRI. Moreover, higher levels of kynurenine and kynurenic acid were observed in the *Kmo^null^* IRI mice compared to the *Kmo*^null^ sham-operated mice. This result may indicate that these metabolites help to protect against AKI after renal IRI, particularly because kynurenic acid has been demonstrated to have protective properties in other inflammatory situations due to its activity at glutamate receptors [95]. 

A 12.5-fold increase in the lysine catabolite saccharopine in IRI kidneys was observed in a recent study examining the differences between renal allograft acute cellular rejection (ACR) and IRI. The findings of this work indicated that the accumulation of saccharopine causes mitochondrial toxicity and may contribute to IRI pathophysiology. Moreover, similar to other reports, increased levels of itaconate and kynurenine were also observed in ACR kidneys. However, the detected changes in metabolites seemed to be unique for IRI and ACR, respectively, indicating that these two conditions have distinct tissue metabolomic signatures [96]. 

Several reports have also demonstrated that IRI can alter the lipidome. For example, Rao et al., evaluated lipid changes in an IRI mouse model using sequential window acquisition of all theoretical spectra-mass spectrometry (SWATH-MS) lipidomics. Their findings indicated that four lipids increased significantly at 6 h after IRI: plasmanyl choline, phosphatidylcholine (PC) O-38:1 (O-18:0, 20:1), plasmalogen, and phosphatidylethanolamine (PE) O-42:3 (O-20:1, 22:2). As anticipated, statistically significant changes were observed in many more lipids at 24 h after IRI. Interestingly, elevated levels of PC O-38:1 persisted at 24 h post-IRI, while renal levels of PE O-42:3 decreased alongside all ether PEs detected by SWATH-MS at this later time point. Overall, the authors found that coupling SWATH-MS lipidomics with MALDI-IMS (Imaging Mass Spectrometry, IMS) for lipid localization provided a better understanding of the role played by lipids in the pathobiology of acute kidney injury [97].

Researchers have also tested whether oxidized phosphatidylcholine (OxPC) molecules are generated following renal IRI. Solati et al., identified fifty-five distinct OxPC molecules in rat kidneys following IRI, including various fragmented (aldehyde and carboxylic-acid-containing species) and nonfragmented products. Among these, 1-stearoyl-2-linoleoyl-phosphatidylcholine (SLPC-OH) and 1-palmitoyl-2-azelaoyl-sn-glycero-3-phosphocholine (PAzPC) were the most abundant after 6 h and 24 h IRI, respectively. The total number of fragmented aldehyde OxPC molecules was significantly elevated in the 6 h and 24 h IRI groups compared to the sham-operated group, while an increase in the level of fragmented carboxylic acid was observed in the 24 h group compared to the sham and 6 h groups. In addition, fragmented OxPC levels were found to be significantly correlated with CR levels [98].

In their recent paper, van Smaalen et al., introduced and employed an interesting new approach based on IMS to rapidly and accurately evaluate acute ischemia in kidney tissue from a porcine model. First, ischemic tissue damage was systematically evaluated by two pathologists; this was followed by the application of MALDI-IMS to study the spatial distributions and compositions of lipids in the same tissues. Whereas the histopathological analysis revealed no significant difference between the tested groups, the MALDI-IMS analysis provided detailed discrimination of severe and mild ischemia based on the differential expression of characteristic lipid-degradation products throughout the tissue. In particular, elevated levels of lysolipids, including lysocardiolipins, lysophosphatidylcholines, and lysophosphatidylinositol, were present after severe ischemia. This data shows IMS’s potential for use in differentiating and identifying early ischemic injury molecular patterns, and as a future tool that can be deployed in kidney assessment [99]. 

Because ischemia and reperfusion are inevitable consequences of kidney transplantation, and because DGF is a manifestation of IRI, Wijermars et al., used kidney transplantation as a clinical model of IRI to evaluate the role of the hypoxanthine-xanthine oxidase (XO) axis in human IRI. The sample group for this study consisted of patients undergoing renal allograft transplantation (n = 40), who were classified into three groups based on the duration of ischemia: short, intermediate, and prolonged. The results of the analysis confirmed the progressive accumulation of hypoxanthine during ischemia. However, differences in arteriovenous concentrations of UA and an in situ enzymography of XO did not indicate relevant XO activity in IRI kidney grafts. Moreover, renal malondialdehyde and isoprostane levels and allantoin formation were assessed during the reperfusion period to determine whether a putative association exists between hypoxanthine accumulation and renal oxidative stress. The absence of the release of these markers indicated the lack of an association between ischemic hypoxanthine accumulation and post-reperfusion oxidative stress. Based on these results, the authors suggest that the hypoxanthine-xanthine oxidase axis is not involved in the initial phase of clinical IRI [100]. In their clinical study, Kostidis et al., employed NMR spectroscopy to analyse the urinary metabolome of DCD transplant recipients at multiple time points in an attempt to identify markers that predict the prolonged duration of functional DGF [79]. To this end, urine samples were collected at 10, 42, 180, and 360 days post-transplantation. Their analysis revealed that samples collected on day 10 had a different profile than samples obtained at the other time points. At day 10, D-glucose, 2-aminobutyrate, valine, p-hydroxyhippurate, fumarate, 2-ethylacrylate, leucine, and lactate were significantly elevated in patients with DGF compared to those without DGF, while asparagine, DMG, 3-hydroxyisobutyrate, 3-hydroxyisovalerate, 2-hydroxy-isobutyrate, and histidine were significantly reduced in the DGF group. Urine samples from patients with prolonged DGF (≥21 days) showed increased levels of lactate and lower levels of pyroglutamate compared to participants with limited DGF (<21 days). Moreover, the ratios of all metabolites were analysed via logistic regression analysis in an attempt to further distinguish prolonged DGF from limited DGF. The results of this analysis showed that the combination of lactate/fumarate and branched chain amino acids (BCAA)/pyroglutamate provided the best outcome, predicting prolonged DGF with an AUC of 0.85. Given these results, the authors concluded that it is possible to identify kidney transplant recipients with DGF based on their altered urinary metabolome, and that it may also be possible to use these two ratios to predict prolonged DGF [79]. 

In another study, Lindeman et al., examined possible metabolic origins of clinical IRI by integrating data from 18 pre- and post-reperfusion tissue biopsies with 36 sequential arteriovenous blood samplings from grafts in three groups of subjects, including LD and DD grafts with and without DGF. The integration of metabolomics data enabled Lindeman et al., to determine a discriminatory profile that can be used to identify future DGF. This profile was characterized by impaired recovery of the high-energy phosphate-buffer, phosphocreatine, in DGF grafts post-reperfusion, as well as by persistent post-reperfusion ATP/GTP catabolism and significant ongoing tissue damage. The impaired recovery of high-energy phosphate occurred despite the activation of glycolysis, fatty acid oxidation, glutaminolysis, autophagia and was found to be related to a defect at the level of the oxoglutarate dehydrogenase complex in the Krebs cycle. Hence, Lindeman et al.’s findings suggest that DGF is preceded by a post-reperfusion metabolic collapse, leading to an inability to sustain the organ’s energy requirements. Thus, efforts aimed at preventing DGF should aim to preserve or restore metabolic competence [101].

### 3.3. New Solutions in Perfusion Control

Organ-preservation technologies have been garnering significant interest for graft quality assessment, advanced organ monitoring, and treating transplanted kidneys during machine perfusion. As mentioned above, SCS and HMP are two of the more common methods of hypothermic preservation applied in clinical settings at present. In SCS, the kidney is submerged in a cold preservation fluid and placed on ice in an icebox; in HMP, a device pumps cold preservation fluid through the renal vasculature, which has been revealed to improve post-transplant outcomes [102]. NMP is another dynamic preservation strategy that involves the circulation of a perfusion solution through the kidney. The NMP conditions are designed to nearly replicate physiological conditions, which makes a real-life assessment of the graft possible prior to transplantation [103,104]. NMP has been recently translated into clinical practice, but this application is still at an experimental stage. However, early clinical results are promising [103,105]. Because preservation/perfusion solutions serve as a non-invasive source for the analysis of biomarkers, numerous studies have employed it for the purposes of graft quality assessment. In this section of this paper, we summarize the latest findings and studies that have used preservation/perfusion fluid and perfusion control in kidney transplantation (Table 1).

Coskun et al., used proteomic techniques to analyse the protein profiles of preservation fluid used in SCS kidneys. Their findings revealed significant correlations between protein levels and donor age (23 proteins), cold ischemia time (5 proteins), recipients’ serum BUN (12 proteins), and CR levels (7 proteins). The identified proteins belonged to groups related to the structural constituent of the cytoskeleton, serine-type endopeptidase inhibitor activity, peptidase inhibitor activity, cellular component organization or biogenesis, and cellular component morphogenesis, among others [106]. In another proteomic study of preservation fluid, five potential biomarkers (leptin, periostin, granulocyte-macrophage colony-stimulating factor (GM-CSF), plasminogen activator inhibitor-1, and osteopontin) were identified in a discovery panel for differentiating kidneys with immediate function from those with DGF. Further analysis yielded a prediction model based on leptin and GM-CSF. Receiver-operating characteristic analysis revealed an AUC of 0.87, and the addition of recipient BMI significantly increased the model’s predictive power, resulting in an AUC of 0.89 [107]. The metabolomic study compared the level of metabolites in perfusate samples collected prior to transplantation, during static cold storage, and between the allografts exhibiting DGF and IGF, while an integrated NMR-based analysis revealed a significant elevation in α-glucose and citrate levels, and significant decreases in taurine and betaine levels in the perfusate of DGF allografts [108]. 

In the last few years, several studies have documented the benefits of HMP over SCS, including improved short-term outcomes and reduced risk of DGF [109,110,111]. However, reports suggesting that HMP improves long-term graft function are inconclusive [102,111]. Some research groups have compared HMP with SCS to evaluate HMP’s potential to improve kidney-graft outcomes [109,112] and to better understand the long-term benefits associated with its use [111,113]. At the same time, other groups have investigated how the use of oxygenated HMP impacts post-transplant outcomes, and how it can be used to further optimize kidney preservation, thereby expanding the number of organs available for transplant [102,114]. Furthermore, perfusion solution has been used in the search for useful biomarkers of graft quality and potential therapeutic targets. The analysis of perfusates from donor after brain death (DBD), DCD, and LD kidneys showed that DCD kidneys contained the highest levels of matrix metalloproteinase-2 (MMP-2), lactate dehydrogenase (LDH), and NGAL, followed by DBD and LD kidneys, respectively, suggesting a greater amount of injury in the DCD kidneys. Moreover, the DCD kidney perfusate contained significantly higher levels of protein compared to the DBD and LD perfusates, with quantitative analysis of the protein spots revealing significant differences between the groups in relation to seven spots: peroxiredoxin-2, FABP, A1AT, heavy chain of immunoglobulin, serum albumin, fragment of collagen 1, and protein deglycase (DJ-1) [115]. In another proteomic study, perfusate analysis of DBD kidneys preserved via HMP was performed to identify differences between the proteomic profiles of kidneys with good (GO) and suboptimal outcomes (SO) one-year post-transplantation. Analysis of samples collected 15 min after the start of HMP (T1) and before the termination of HMP (T2) indicated that the 100 most abundant proteins demonstrated discrimination between grafts, with a GO and SO at T1. Increased proteins were involved in classical complement cascades at both T1 and T2, while a decreased abundance of lipid metabolism at T1 and cytoskeletal proteins at T2 in GO (vs. SO) was also observed. Perfusate analysis at T1 revealed a predictive value of 91% for ATP-citrate synthase and fatty acid-binding protein 5, and analysis at T2 showed a predictive value of 86% for immunoglobulin heavy variable 2–26 and desmoplakin. In summary, HMP perfusate profiles for DBD kidneys can distinguish between outcomes one-year post-transplantation, providing a potential non-invasive method of assessing donor organ quality [2]. 

MicroRNAs in kidney machine perfusion solutions have also been considered as new biomarkers for graft function. For instance, Gómez dos Santos et al., conducted a prospective cohort study to investigate graft dysfunction in kidney transplantation from ECD. To this end, they employed a mean expression value approach, which confirmed the significance of a subset of the miRNAs previously identified with the development of delayed graft function, namely, miR-486-5p, miR-144-3p, miR-142-5p, and miR-144-5p. These results confirmed that perfusion fluid can be a valuable pre-transplantation source of organ-viability biomarkers [116]. 

In another study, Tejchman et al., assessed oxidative stress markers from the hypothermic preservation of transplanted kidneys. In particular, they sought to analyse the activity of enzymes and levels of non-enzymatic compounds involved in antioxidant defense mechanisms. These compounds, which included glutathione (GSH), glutathione peroxidase (GPX), catalase (CAT), superoxide dismutase (SOD), glutathione reductase (GR), glutathione transferase (GST), thiobarbituric acid reactive substances (TBARS), malondialdehyde (MDA), were measured in preservation solutions before the transplantation of human kidneys grafted from DBD. The study group was divided into two groups based on the method of kidney storage, with Group 1 consisting of HMP kidneys (n = 26) and Group 2 consisting of SCS kidneys (n = 40). There were aggregations of significant correlations between kidney function parameters after KTx and oxidative stress markers, namely: diuresis and CAT; Na^+^ and CAT; K^+^ and GPX; and urea and GR. Moreover, there were aggregations of correlations between recipient blood count and oxidative stress markers, including CAT and monocyte count; SOD and white blood cell count; and SOD and monocyte count. However, there was an issue of unequivocal interpretation because none of the observed aggregations constituted conditions that supported the authors’ hypothesis that kidney function after KTx can be predicted based on oxidative stress markers measured during preservation. Moreover, it would be hard to conclude that the blood count alterations observed in the repeated measurements after KTx were unrelated to factors other than oxidative stress or acidosis. As the authors suggest, many other factors may modify blood count, including operative stress, bleeding, immunosuppression, and microaggregation [117].

Longchamp et al., presented an interesting and non-invasive method of assessing graft quality during perfusion based on the use of ^31^P pMRI spectroscopy to detect high-energy phosphate metabolites, such as ATP. Thus, pMRI can be used to predict the energy state of a kidney and its viability before transplantation. In addition, Longchamp et al., also performed gadolinium perfusion sequences, which allowed them to observe the internal distribution of the flow between the cortex and the medulla. pMRI showed that warm ischemia caused a reduction in ATP levels, but not its precursor, adenosine monophosphate (AMP). Moreover, they found that ATP levels and cortical and medullary gadolinium elimination were inversely correlated with the severity of kidney histological injury. Thus, the measured parameters may be considered as biomarkers of kidney injury after warm ischemia, and Longchamp et al.’s method provides an innovative non-invasive approach to assessing kidney viability prior to transplantation [118].

Other researchers have examined whether a correlation exists between the level of extracellular histones in machine perfusates and the viability of DD kidneys. Extracellular histone levels were significantly elevated in the perfusates of kidneys with post-transplant graft dysfunction, and they were considered an independent risk factor for DGF and one-year graft failure, but not for PNF. One-year graft survival was 12% higher in the low-histone-concentration group (*p* = 0.008) compared to the higher-histone-concentration group. Hence, the quantitation of extracellular histones might contribute to the evaluation of post-transplant graft function and survival [119].

NMP is an emerging approach for donor organ preservation and functional improvements in kidney transplantation. However, methods for evaluating organs via NMP have yet to be developed, and the development of novel graft quality assessment solutions has only recently come into focus. 

Kaths et al., used a porcine model to investigate whether NMP is suitable for graft quality assessment prior to transplantation. They found that intra-renal resistance was lowest in the HBD group and highest in the severely injured DCD group (60 min of warm ischemia), and that the initiation of NMP was correlated with post-operative renal function. Markers of acid-base homeostasis (pH, HCO_3_^–^, base excess) correlated with post-transplantation renal function. Furthermore, concentrations of lactate and aspartate aminotransferase were lowest in perfusate from non-injured grafts (vs. DCD kidneys) and were correlated with post-transplantation kidney function. Kaths et al., found that perfusion characteristics and clinically available perfusate biomarkers during NMP were correlated with post-transplantation kidney graft injury and function. However, further research is needed to identify perfusion parameter thresholds for DGF and PNF [120]. 

HSI combined with NMP was introduced as a novel approach for monitoring physiological kidney parameters. The experimental results of an HSI-based oxygen-saturation calculation indicated that HSI is useful for monitoring oxygen saturation distribution and identifying areas with a reduced oxygen supply prior to transplantation. Moreover, camera-based measurements are easy to integrate with a perfusion setup and allow the fast and non-invasive measurement of tissue characteristics [121]. Subsequent research has explored how to improve algorithms for determining kidney oxygen saturation [122]. Unfortunately, the application of HSI is limited by the propagating light’s low penetration depth, which makes it impossible to detect deeper tissue injuries. However, based on the fact that most metabolic activity occurs in the kidney cortex, the combined use of HSI and NMP offers a promising and easy-to-use method for assessing the status of the organ and for chemical imaging [121,122].

Hyperpolarized MRI and spectroscopy (MRS) using pyruvate and other ^13^C-labeled molecules offers a novel approach to monitoring the state of ex vivo perfused kidneys. In one study, the state of a porcine kidney was quantified using acquired anatomical, functional, and metabolic data. The findings showed an apparent reduction in pyruvate turnover during renal metabolism compared with the typical in vivo levels observed in pigs, while perfusion and blood gas parameters were found to be in the normal ex vivo range. Mariager et al.’s findings demonstrate the applicability of these techniques for monitoring ex vivo graft metabolism and function in a large animal model that resembles human renal physiology [123]. 

In another study, researchers sought to investigate the link between the urinary biomarkers, endothelin-1 (ET-1), NGAL, and KIM-1, and NMP parameters in order to improve kidney assessment prior to transplantation. Fifty-six kidneys from DD were used in this work, with each kidney being subjected to 1 h of NMP, followed by assessment based on macroscopic examination, renal blood flow, and urine output. The levels of ET-1 and NGAL measured in the urine samples after 1 h of NMP were significantly associated with perfusion parameters during NMP. These biomarkers and NMP perfusion parameters were also significantly associated with terminal graft function in the donor. However, KIM-1 was not correlated with the perfusion parameters or the donor’s renal function. Larger studies are required to determine the usefulness of using these biomarkers with NMP to predict transplant outcomes. Despite this limitation, this study undoubtedly demonstrates that measuring urinary biomarkers during NMP provides additional information about graft quality [124].

**Table 1:** Emerging trends in donor graft quality assessment techniques.

Application	Category	Model	Type of Sample	Main Conclusions	Author
Evaluation of gene expression profile of kidney submitted to ischemic injury	Donor graft quality	Pig	Tissue	ischemia leads to the full reprogramming of the transcriptome of major pathways such those related to oxidative stress responses, cell reprogramming, cell-cycle, inflammation and cell metabolism	Giraud et al. [55]
Investigation of the features of perirenal adipose tissue as an indicator of the detrimental impact of the ECD microenvironment on a renal transplant	Donor graft quality	Human	Perirenal adipose tissue	↑ genes associated with the inflammatory response, cytokine secretion, and circulatory system development ↓ genes associated with regulating metabolic processes and regulating the circulatory system development	Boissier et al. [56]
Evaluation of donor category influence on borderline changes in kidney allografts by molecular fingerprints	Donor graft quality	Human	Tissue	early borderline changes in ECD kidneys were characterized by the most increased regulation of inflammation, extracellular matrix remodeling, and AKI transcripts compared to SCD and LD groups	Hruba et al. [57]
Exploration of the association between plasma mtDNA levels and post-transplant renal allograft function	Donor graft quality	Human	Plasma	plasma mtDNA may be a non-invasive predictor of DGF and allograft function at 6 months after transplantation, and it also correlates with allograft survival mtDNA may serve as a surrogate predictive marker for PNF	Han et al. [58]
Searching for urinary miRs that can be a biomarker for AKI	IRI	Mouse Human	Urine; Tissue Urine; Serum	urinary miR-16 may serve as a valuable indicator for AKI patients	Chen et al. [59]
Determination of the role of miR-17- 92 in IRI-induced AKI	IRI	Mouse	Tissue	overexpression of miR-17-92 may antagonize the side-effects of IRI on the proximal tubules in vivo	Song et al. [61]
Investigation of the expression of renal miRNAs following renal IRI	IRI	Rat	Tissue	↑ miR- 27a downregulated the expression of TLR 4, which resulted in inhibition of inflammation, cell adhesion and cell death in IRI	Wang et al. [62]
Identification of candidate genes involved in renal IRI	IRI	Mouse	Tissue	IRI induces changes in the expression of SPRR2F, SPRR1A, MMP-10, *Malat1*, and *miR-139-5p* in the kidney, suggesting the utility of this panel as a biomarker of the renal IRI	Su et al. [64]
Examination of a link between activation of IL-33 transcription by BRG1 in endothelial cells and renal IRI	IRI	Mouse	Tissue	endothelial BRG1 deficiency alleviates renal inflammation following IRI in mice with a concomitant reduction in IL-33 levels	Liu et al. [65]
Screening for differentially expressed genes in renal IR-injured mice using a high-throughput assay	IRI; DGF	Mouse Human	Tissue, Serum Plasma	plasma Corin was downregulated in kidney transplantation recipients complicated with DGF Corin might be a potential biomarker that is associated with DGF of kidney transplantation	Hu et al. [67]
Unbiased urinary microRNA profiling to identify DGF predictors after kidney transplantation.	DGF	Human	Urine	combined measurement of six microRNAs (miR-9, mIR-10a, miR-21, miR-29a, miR-221, miR-429) had predictive value for DGF following KT	Khalid et al. [68]
High-throughput sequencing to expression profiling of exosomal miRNAs obtained from the peripheral blood of patients with DGF	DGF	Human	Plasma	↑ hsa-miR-33a-5p R-1, hsa-miR-98-5p, hsa-miR-151a-5p in kidney recipients with DGF	Wang et al. [69]
Examination of miR-146a-5p expression in kidney transplant recipients with DGF	DGF	Human	Tissue; Whole blood	miR-146a-5p expression has a unique pattern in the renal tissue and perhaps in a blood sample in the presence of DGF	Milhoransa et al. [71]
Evaluation of PBMC TLR4 expression of renal graft recipients with DGF	DGF	Human	Tissue; Whole blood	low TLR4 expression in patients with DGF may be related to a poor prognosis for graft capability analysis of TLR4 expression change may be a valuable parameter for the evaluation of immunosuppression effectiveness	Zmonarski et al. [72]
Profiling of molecular changes associated with decreased resilience and impaired function of human renal allografts	DGF	Human	Tissue	identified 42 transcripts associated with IFNγ signaling, which in allografts with DGF exhibited a greater magnitude of change in transcriptional amplitude and higher expression of noncoding RNAs and pseudogenes identified	McGuinness et al. [3]
Searching for urinary biomarkers that predict reduced graft function after DD kidney transplantation	RGF	Human	Urine	utility of donor urinary NGAL, KIM-1, L-FABP levels in predicting RGF the model including donor urinary NGAL, L-FABP, and serum CR showed a better predictive value for RGF than donor serum CR alone	Koo et al. [74]
Evaluation of associations between DD urine injury biomarkers and kidney transplant outcomes	DGF	Human	Urine	higher urinary NGAL and L-FABP levels correlated with slightly decreased 6-month eGFR only among patients without DGF donor urine injury biomarkers correlate with donor AKI but have poor predictive value for outcomes in kidney transplant recipients	Reese et al. [75]
Assessment of C3a and C5a in urine samples as biomarkers for post-transplant outcomes	DGF	Human	Urine	urinary C5a was associate with the degree of donor AKI in the absence of clinical donor AKI, donor urinary C5a concentrations associate with recipient DGF	Schröppel et al. [76]
Assessment of urinary and perfusate concentrations of MCP-1 from kidneys on HMP as an organ function indicator	AKI; DGF	Human	Urine; Perfusate	higher concentrations of uMCP-1 are independently associated with donor AKI donor uMCP-1 concentrations were modestly associated with higher recipient six-month eGFR in those without DGF donor uMCP-1 has low clinical utility due to the lack of correlation with graft failure	Mansour et al. [77]
Evaluation of the proteome of suEVs and its changes throughout LD transplantation	Donor graft quality	Human	Urine; Tissue	the abundance of PCK2 in the suEV proteome 24 h after transplantation may have a predictive value for overall kidney function one year after transplantation	Braun et al. [80]
Proteomic study of differentially expressed proteins in BD rabbits kidneys	Donor graft quality	Rabbit	Tissue; Serum	the results indicated alterations in levels of several proteins in the kidneys of those with BD, even if the primary function and the structural changes were not obvious PHB may be a novel biomarker for primary quality evaluation of kidneys from DBD	Li et al. [81]
Investigation of the influence of BD on systemic and specifically hepatic and renal metabolism in a rodent BD model	Donor graft quality	Rat	Plasma; Urine; Tissue	the kidneys undergo metabolic arrest and oxidative stress, turning to anaerobic energy generation as renal perfusion diminishes	Van Erp et al. [82]
Unbiased integrative proteo-metabolomic study in combination with mitochondrial function analysis of kidneys exposed to IRI to investigate its effects at the molecular level	IRI	Rat	Tissue	proteins belonging to the acute phase response, coagulation and complement pathways, and FA signaling were elevated after IRI metabolic changes showed increased glycolysis, lipids, and FAs after 4 h reperfusion mitochondrial function and ATP production were impaired after 24 h	Huang et al. [83]
Integrative proteome analysis of potential and predominantly renal injury biomarkers considering changes occurring in the tissue and echo in serum and urine protein profiles	IRI	Pig	Serum; Urine; Tissue	four urinary proteins with primarily renal gene expression were changed in response to managed kidney IRI and may be biomarkers of kidney dysfunction: aromatic-L-amino-acid decarboxylase (AADC), S-methylmethionine–homocysteine S-methyltransferase BHMT2 (BHMT2), cytosolic beta-glucosidase (GBA3), and dipeptidyl peptidase IV (DPPIV)	Malagrino et al. [84]
Evaluation of the changes in the proteome of kidney subjected to ischemia during machine cold perfusion with doxycycline	IRI	Rat	Tissue; Perfusate	analysis showed a significant difference in 8 enzymes, all involved in cellular and mitochondrial metabolism N(G),N(G)-dimethylarginine dimethylaminohydrolase and phosphoglycerate kinase 1 were decreased by cold perfusion, and perfusion with Doxy led to an increase in their levels	Moser et al. [85]
Proteomics analysis determinating the molecular differences between NMP human kidneys with URC and UR	IRI	Human	Tissue	NMP with URC permits prolonged preservation and revitalizes metabolism to possibly better cope with IRI in discarded kidneys	Weissenbacher et al. [86]
TUPA to identify protein biomarkers of delayed recovery following KTx	DGF	Human	Urine	C4b-binding protein alpha chain, serum amyloid P-component, Guanylin, and Immunoglobulin Super-Family Member 8 were identified that together distinguished DGF with a sensitivity of 77.4%, specificity of 82.6%	Williams et al. [87]
Assessment of the diagnostic and prognostic role of NGAL in DGF and chronic allograft nephropathy	DGF	Human	Serum; Urine	high levels of NGAL characterized DGF patients since the first day after transplantation in urine and serum urine NGAL presented a better diagnostic profile than serum NGAL	Lacquaniti et al. [88]
Investigation of changes of urinary TIMP-2 and IGFBP7 in the first days after KTx and their diagnostic utility for predicting DGF outcomes	DGF	Human	Urine	urinary TIMP-2, but not IGFBP7, is a potential biomarker to predict the occurrence and duration of DGF in DCD kidney transplant recipients	Bank et al. [89]
Investigation of organ-specific metabolic profiles of the liver and kidney during BD and afterwards during NMP of the kidney	Donor graft quality	Rat	Tissue; Plasma; Urine	immediately following BD induction, BD animals demonstrated significantly increased lactate levels, and after 4 h of BD, alanine production decreased in the kidney during IPK perfusion, renal glucose oxidation was decreased following BD vs sham animals	van Erp et al. [90]
Investigation of the acute and prolonged metabolic consequences associated with IRI, and elucidation whether the early injury mediated metabolic reprogramming can predict the outcome of the injury	IRI	Rat	Tissue; Plasma	significant correlation between the intra-renal metabolic profile 24 h after reperfusion and 7 d after injury induction identifying the balance between the anaerobic and aerobic metabolism with the use of hyperpolarized ^13^C-labeled pyruvate has a great potential to be used in the future as a prognostic biomarker	Nielsen et al. [91]
NMR identification of metabolic alterations to the kidney following IRI	IRI	Mouse	Urine; Serum; Tissue	higher levels of valine and alanine and decreased metabolites such as trigonelline, succinate, 2-oxoisocaproate, and 1-methyl-nicotinamide were found in urine following IRI due to altered kidney function or metabolism	Chihanga et al. [92]
Monitoring of the effect of oxidative stress and ischemia on the condition of kidneys using SPME-LC-HRMS platform	Organ ischemia	Rabbit	Tissue	pronounced alterations in metabolic profile in kidneys induced by ischemia and oxidative stress as a cold storage function were reflected in levels of essential amino acids and purine nucleosides	Stryjak et al. [93]
Assessment of the role of kynurenine 3-monooxygenase as an essential regulator of renal IRI	IRI	Mouse	Plasma; Urine; Tissue	KMO is highly expressed in the kidney and exerts major metabolic control over the biologically active kynurenine metabolites 3-hydroxykynurenine, kynurenic acid, and downstream metabolites mice lacking functional KMO kept renal function, decreased renal tubular cell injury, and fewer infiltrating neutrophils compared with control mice	Zheng et al. [95]
Unbiased tissue metabolomic profiling of IRI and ACR in murine models to identify novel biomarkers and to provide a better understanding of the pathophysiology	IRI; ACR	Mouse	Tissue	the lysine catabolite saccharopine 12.5-fold was increased in IRI kidneys and caused mitochondrial toxicity itaconate and kynurenine increased levels were found in ACR kidneys	Beier et al. [96]
Detection of early lipid changes in AKI using SWATH lipidomics coupled with MALDI tissue imaging	IRI	Mouse	Tissue	increase in plasmanyl choline, phosphatidylcholine (PC) O-38:1 (O-18:0, 20:1), plasmalogen, and phosphatidylethanolamine (PE) O-42:3 (O-20:1, 22:2) concentrations at 6 h after IRI PC O-38:1 elevations were maintained at 24 h post-IR, while renal PE O-42:3 levels reduced, as were all ether PEs detected by SWATH-MS at this later time point	Rao et al. [97]
Determination of the individual OxPC molecules generated during renal IRI	IRI	Rat	Tissue	SLPC-OH and PAzPC were the most abundant OxPC species after 6 h and 24 h IRI, respectively total fragmented aldehyde OxPC were significantly elevated in IRI groups than sham groups fragmented carboxylic acid elevated in 24 h group compared with other groups	Solati et al. [98]
Rapid identification of IRI in renal tissue by Mass-Spectrometry Imaging	IRI	Pig	Tissue	MALDI-IMS provided of detailed discrimination of severe and mild ischemia by differential expression of characteristic lipid-degradation products throughout the tissue lysolipids, including lysocardiolipins, lysophosphatidylcholines, and lysophosphatidylinositol were elevated after severe ischemia	Van Smaalen et al. [99]
Evaluation of the involvement of the hypoxanthine-XO axis in the IRI that occurs during kidney transplantation	IRI	Human	Plasma; Tissue	arteriovenous concentration differences of UA and in situ enzymography of XO did not indicate significant XO activity in IRI kidney grafts absent release of malondialdehyde, isoprostane and allantoin is not consistent with an association between ischemic hypoxanthine accumulation and postreperfusion oxidative stress	Wijermars et al. [100]
Prediction of prolonged duration of DGF in DCD kidney transplant recipients by urinary metabolites profiling	DGF	Human	Urine	the metabolites associated with prolonged DGF are handled by proximal tubular epithelial cells and reflect tubular (dys)function lactate/fumarate and BCAAs/pyroglutamate ratios were useful to predict prolonged duration of DGF	Kostidis et al. [79]
Explorative metabolic assessment based on an integrated, time-resolved strategy involving sequential evaluation of AV differences over reperfused grafts and parallel profiling of graft biopsies	DGF	Human	Tissue; Plasma	DGF is preceded by a post-reperfusion metabolic collapse, leading to an inability to sustain the organ’s energy requirements	Lindeman et al. [101]
Analysis of the proteins and peptides that are passed from the kidneys to the preservation fluid during organ preservation	Perfusion control	Human	Preservation fluid	the relevant correlations between the levels of proteins and donors’ age (23 proteins), cold ischemia time (5), recipients’ serum BUN (12), and CR (7) levels were observed identified proteins belonged to groups related to the structural constituent of the cytoskeleton, serine-type endopeptidase inhibitor activity, peptidase inhibitor activity, cellular component organization or biogenesis, and cellular component morphogenesis	Coskun et al. [106]
Searching for proteins accumulating in preservation solutions during SCS as biomarkers to predict posttransplantation graft function	Perfusion control	Human	Preservation fluid	five potential biomarkers (leptin, periostin, GM-CSF, plasminogen activator inhibitor-1, and osteopontin) were identified in a discovery panel, differentiating kidneys with IGF versus DGF prediction model based on leptin and GM-CSF and recipient BMI showed an AUC of 0.89	van Balkom et al. [107]
Analysis of perfusates during SCS to obtain the metabolite profiles of DGF and IGF allografts	Perfusion control	Human	Preservation fluid	significant elevation in α-glucose and citrate levels and significant decreases in taurine and betaine levels in the perfusate of DGF allografts	Wang et al. [108]
Proteomic study of perfusate from HMP of transplant kidneys	Perfusion control	Human	Perfusate	the highest levels of MMP-2, LDH, and NGAL were seen for the DCD kidneys, followed by the DBD kidneys and then LD total protein in the perfusate from DCD was significantly increased than that in the perfusate from other donors	Moser et al. [115]
Proteomic perfusate analysis of DBD kidneys preserved using HMP to identify the differences between proteomic profiles of kidneys with a good and suboptimal outcome	Perfusion control	Human	Perfusate	DBD kidney HMP perfusate profiles can distinguish between outcome one year after transplantation increased proteins involved in classical complement cascades and a decreased levels of lipid metabolism at T1 and cytoskeletal proteins at T2 in GO versus SO were observed	van Leeuwen et al. [2]
Evaluation of miRNAs in kidney machine perfusion fluid as novel biomarkers for graft function	Perfusion control	Human	Perfusate	confirmation of the significance of a subset of the miRNAs previously identified for DGF development and composed of miRNAs miR-486-5p, miR-144-3p, miR-142-5p, and miR-144-5p	Gómez-Dos-Santos et al. [116]
Influence of method of kidney storage on oxidative stress and post-transplant kidney function parameters	Perfusion control	Human	Perfusate; Whole blood	correlations between kidney function parameters after KTx and oxidative stress markers: diuresis or Na^+^ and CAT, K^+^ and GPX, urea and GR were found	Tejchman et al. [117]
Ex vivo evaluation of kidney graft viability during perfusion using ^31^P MRI spectroscopy	Perfusion control	Pig	n.a.	warm ischemia induced significant histological damages, delayed cortical and medullary Gadolinium elimination (perfusion), and decreased ATP levels, but not AMP ATP levels and kidney perfusion are both inversely linked to the degree of kidney histological damage	Longchamp et al. [118]
Assessment of an association between the presence of extracellular histones in machine perfusates and deceased donor kidney viability	Perfusion control	Human	Perfusate	extracellular histone concentrations were significantly higher in perfusates of kidneys with posttransplant graft dysfunction and were an independent risk factor for DGF and one-year graft failure, but not for primary nonfunction	van Smaalen et al. [119]
Organ quality assessment during NMP	Perfusion control	Pig	Perfusate; Whole blood; Urine	intra-renal resistance was lowest in the HBD group and highest in the severely injured DCD group and at the initiation of NMP correlated with postoperative renal function markers of acid-base homeostasis, lactate and aspartate aminotransferase perfusate concentrations were correlated with post-transplantation renal function	Kaths et al. [120]
Hyperpolarized MRI and spectroscopy using pyruvate and other 13C-labeled molecules as a novel tool for monitoring the state of ex vivo perfused kidneys	Perfusion control	Pig	n.a.	renal metabolism displayed an apparent reduction in pyruvate turnover compared with pigs’ usual in vivo levels perfusion and blood gas parameters were in the normal ex vivo range	Mariager et al. [123]
Examination of the relationship between urinary biomarkers and NMP parameters in a series of human kidneys	Perfusion control	Human	Urine; Serum	urinary ET-1 and NGAL assessed after 1 h of NMP were significantly associated with perfusion parameters during NMP and terminal renal function in the donor KIM-1 was not linked with perfusion parameters or donor’s renal function	Hosgood et al. [124]

↑—increase of expression; ↓—decrease of expression; n.a—not applicable.

## 4. Conclusions

New diagnostic solutions for accurately assessing renal graft quality are needed to improve the process for selecting suitable donors, more efficiently managing complications, and prolonging graft survival. Rapid advances in imaging, omics technology, and perfusion methods have led to the development of a wide range of new tools and biomarkers that could be applied to evaluate graft quality. Unfortunately, most of the methods mentioned in the review are based on animal models or require sophisticated technology with a long turn-around time to obtain the results, which significantly limits their potential for clinical use in the form of rapid commercial tests at present. However, non-invasive solutions, including imaging and the measurement of biomarkers in urine, blood, and perfusion fluid, appear to be promising with respect to their ability to be translated to a clinical setting. These studies include mtDNA and miRNAs determination based on commercially available kits for the isolation of genetic material in combination with the RT-PCR technique widely used in laboratory practice. A similar clinical potential is demonstrated by the determination of biomarkers such as NGAL, KIM-1, L-FABP and C5a in urine by ELISA, also routinely used in diagnostics. Nevertheless, the translation of biomarkers from the discovery stage to clinical practice is still challenging due to the complex and multifactorial type of injuries, the absence of standard guidelines for method validation, and adequate prospective and retrospective cohort studies. Larger, multi-centre validation studies are needed before new solutions can be widely implemented in clinics. Moreover, it will be imperative for future research to explore new technologies and integrate molecular measurements from large data sets reported in different experiments.

## Data Availability

No new data were created or analyzed in this study. Data sharing is not applicable to this article.

## Abbreviations

ACR	acute cellular rejection
AKI	acute kidney injury
AMP	adenosine monophosphate
AR	acute rejection
ASL	arterial spin labelling
ATP	adenosine triphosphate
AUC	area under the curve
BCAA	branched chain amino acids
BD	brain death
BMI	body mass index
BUN	blood urea nitrogen
CAT	catalase
CR	creatinine
CTA	computed tomography
CTA	computed tomography angiography
DBD	donor after brain death
DCD	donor after cardiac death
DD	deceased donors
DEG	Differentially expressed genes
DGF	delayed graft function
DJ-1	protein deglycase
Doxy	doxycycline
ECD	expanded criteria donors
eGFR	estimated glomerular filtration rate
ET-1	endothelin-1
FA	fatty acid
FABP	fatty acid-binding protein
FAO	fatty acid oxidation
FC	fold change
FS	frozen section
GM-CSF	Granulocyte-macrophage colony-stimulating factor
GO	good outcome
GPX	glutathione peroxidase
GR	glutathione reductase
GSH	glutathione
GST	glutathione transferase
GTP	guanosine triphosphate
HMP	Hypothermic machine perfusion
HSI	Hyperspectral Imaging
ICG	indocyanine green
IGF	immediate graft function
IGFBP7	insulin-like growth factor binding protein-7
IL-18	Interleukin-18
IPK	normothermic isolated perfused kidney
IRI	ischemia-reperfusion injury
KATs	kynurenine aminotransferases
KDPI	Kidney Donor Profile Index
KDRI	Kidney Donor Risk Index
KIM-1	kidney injury molecule-1
KMO	kynurenine 3-monooxygenase
KTx	Kidney transplantation
LD	living donor
LDH	lactate dehydrogenase
LDKT	living donor kidney transplantation
L-FABP	L-type fatty acid binding protein
lncRNA	long noncoding RNA
MALDI-IMS	Matrix Assisted Laser Desorption/Ionization-Imaging Mass Spectrometry
MALDI-TOF-MS	Matrix Assisted Laser Desorption/Ionization Time-of-Flight Mass Spectrometry
MCP-1	Monocyte chemoattractant protein-1
MDA	malondialdehyde
miRNA/miR	microRNA
MMP-2	matrix metalloproteinase-2
MR	magnetic resonance
MRI	magnetic resonance imaging
MRS	magnetic resonance spectroscopy
MS	mass spectrometry
MSI	mass spectrometry imaging
mtDNA	mitochondrial DNA
NB	needle biopsy
NGAL	neutrophil gelatinase-associated lipocalin
NMP	normothermic machine perfusion
NMR	nuclear magnetic resonance
OxPC	oxidized phosphatidylcholine
PBMC	peripheral blood mononuclear cells
PC	phosphatidylcholine
PCK2	phosphoenol pyruvate carboxykinase
PE	phosphatidylethanolamine
PET	positron emission tomography
PHB	prohibitin
pMRI	^31^P magnetic resonance imaging
PNF	primary nonfunction
PRAT	perirenal adipose tissue
PS	paraffin sections
RGB	Red Green Blue (colour model)
RGF	reduced graft function
RR	renal resistance
RT-PCR	real-time polymerase chain reaction
SCD	standard criteria donors
SCS	Static cold storage
SO	suboptimal outcome
SOD	superoxide dismutase
SPME	solid-phase microextraction
suEVs	small urinary extracellular vesicles
SVF	stromal vascular fraction
SWATH-MS	sequential window acquisition of all theoretical spectra-mass spectrometry
TBARS	thiobarbituric acid reactive substances
TCA	tricarboxylic acid
TIMP-2	tissue inhibitor of metalloproteinases-2
TLR4	Toll-like receptor 4
TUPA	Targeted Urine Proteome Assay
UA	uric acid
UR	urine replacement
URC	urine recirculation
WB	wedge biopsy
WI	warm ischemia
WIT	warm ischemia time
XO	hypoxanthine-xanthine oxidase
